# Novel Blend for Producing Porous Chitosan-Based Films Suitable for Biomedical Applications

**DOI:** 10.3390/membranes8010002

**Published:** 2018-01-03

**Authors:** Norhan Nady, Sherif H. Kandil

**Affiliations:** 1 Chemical and Petrochemicals Engineering Department, Energy, Egypt-Japan University for Science and Technology, Alexandria 21934, Egypt; 2 Polymeric Research Department, Advanced Technology and New Materials Research Institute (ATNMRI), City of Scientific Research and Technological Applications (SRTA-City), Alexandria 21934, Egypt; 3 Materials Science Department, Institute of Graduate Studies and Research, Alexandria University, Alexandria 21526, Egypt; s.kandil@usa.net

**Keywords:** chitosan–gelatin–ferulic acid blend, ferulic acid crosslinker, hexagonal porous film, biodegradable blend, biomedical film/membrane

## Abstract

In this work, a chitosan–gelatin–ferulic acid blend was used in different ratios for preparing novel films that can be used in biomedical applications. Both acetic and formic acid were tested as solvents for the chitosan–gelatin–ferulic acid blend. Glycerol was tested as a plasticizer. The thickness, mechanical strength, static water contact angle and water uptake of the prepared films were determined. Also, the prepared films were characterized using different analysis techniques such as Fourier transform infrared spectroscopy (FT-IR) analysis, X-ray diffraction (XRD), thermal gravimetric analysis (TGA), differential scanning calorimetry (DSC) and scanning electron microscopy (SEM). Acetic acid produced continuous compact surfaces that are not recommended for testing in biomedical applications. The plasticized chitosan–gelatin–ferulic acid blend, using formic acid solvent, produced novel hexagonal porous films with a pore size of around 10–14 µm. This blend is recommended for preparing films (scaffolds) for testing in biomedical applications as it has the advantage of a decreased thickness.

## 1. Introduction

Recently, many natural polymeric materials have been recommended for use in treating the loss or failure of organs and tissues [1]. In a typical therapy, cells are seeded into a temporary polymeric matrix and are allowed to remodel the matrix for a particular desired biomedical application [2,3]. This film, membrane, or polymer-based matrix provides a biodegradable support for cells to grow and regenerate a new organ or tissue. For an ideal matrix material, there are several properties that should be considered, including biocompatibility, microstructure, mechanical strength, degradation rate, the ability to support cell attachment, growth, proliferation, differentiation, retention of the metabolic functions and inhibition of infections. Nature offers a huge set of polymeric materials with great potential to be used as a porous polymeric matrix or film/membrane such as chitosan, starch, gelatin, collagen and so forth [3,4].

Chitosan is a polysaccharide of marine origin consisting of glucosamine and *N*-acetyl glucosamine with a β-(1–4) link. It can be obtained from naturally occurring chitin through *N*-deacetylation (i.e., treatment with an alkali at elevated temperature) [1]. The solubility of chitosan in an acidic medium (i.e., a pH <6.5, which can convert glucosamine units into the soluble form R–NH_3_^+^) depends on its content: the amount of free amino and *N*-acetyl groups. Chitosan is known for its antimicrobial properties against fungi and bacteria. This is attributed to the cationic nature of the amino groups in chitosan that are associated with the anions in the bacterial cell walls. Their association suppresses biosynthesis and disrupts mass transport across the cell wall leading to the death of bacteria cells [2,5]. Other researchers [6] proposed that chitosan of a low molecular weight is capable of penetrating the cell’s nucleus itself, interacting with the DNA, which suppresses the synthesis of proteins and inhibits cell growth. Chitosan has other numerous merits such as nontoxicity, biodegradability, biocompatibility, heavy metal adsorption effect, antioxidation effect, film formability and bioadhesive characteristics [1,3,4,5]. However, the drawbacks are limited mechanical properties, processability and variability between different batches, all of which hinder expanding applications of chitosan [4,7]. Therefore, the combination of chitosan and other natural polymers [8,9], synthetic polymers [10], blending with inorganic molecules [11,12], or both organic and inorganic [13] and/or the addition of plasticizing agents [14] have been targeted to improve the aforementioned drawbacks. Chitosan can also form complexes with crosslinking agents such as glutaraldehyde [15,16], which makes chitosan a useful polymer for artificial membranes, scaffolds, sponges, nanoparticles, beads, or microspheres and so forth [17,18,19,20]. Chitosan scaffolds have been produced using different techniques including freeze-drying [21], solvent-exchange/phase-separation [22], gelation of a solution of chitosan using an alkaline solution below its gelation point [23,24], wet (electro)spinning [25], particle aggregation [26], compression molding followed by salt leaching [27], melt spinning [28,29] and fiber bonding [30,31,32].

Crosslinking is an effective tool for amplifying the characteristics of chitosan-based biomaterials. Typically, the mechanical strength and biostability can be enhanced by crosslinking treatment [1,2,33]. The available amino and hydroxyl groups on chitosan are active sites that have the ability to form different linkages, including amide, ester and/or Schiff base formation [34,35]. Among the huge range of available crosslinkers, ferulic acid seems a very promising candidate for biomedical applications. Ferulic acid is a naturally occurring phenolic compound that has been extensively used in food, cosmetics and pharmaceutical industries owing to its antioxidant [36], antimicrobial [37,38] and anticancer [39] effectiveness. Ferulic acid has been grafted onto chitosan through a carbodiimidemediated coupling reaction [40]. The ferulic acid grafted chitosan showed a reduced crystallinity (by 10%), a decreased decomposition temperature (by about 55 °C) and an improved radical scavenging activity (by 55%) compared to pure chitosan [40]. Also, incorporation of ferulic acid was found to improve the barrier properties and tensile strength of the starch–chitosan blend films [41]. The interaction between the amide and hydroxyl groups of starch and ferulic acid was reported [41,42]. Also, the green starch–chitosan blend films crosslinked by ferulic acid has been investigated for using in gas separation [43].

Gelatin is a protein that is obtained through a controlled denaturing of collagen, the major component of skin, bone and connective tissue [44]. However, bovine and porcine wastes are the most frequent sources of gelatin in addition to fish [45]. Generally, gelatin is known for its film-forming ability but gelatin films have very bad mechanical properties, which limits their application [46]. Chemical or enzymatic treatments [47]; mixing with other natural polymers such as fatty acids [48], oils [49] and chitosan [50]; or mixing with inorganic materials [51] are all previously reported methods that take advantage of the pure component of gelatin. Also, the gelatin film that was cross-linked using ferulic acid exhibited improved strength and a decreased swelling ratio without affecting the water vapor permeability [52].

A chitosan–gelatin polyelectrolyte complex hydrogel membrane that shows improved tensile strength and elongation in the swollen state has been reported [53]. These improved properties were attributed to the electrostatic interaction and hydrogen bonding between the amino polysaccharide and gelatin [54,55]. Foam [56], 3D printed substrates [57,58], electrospinning [59], phase separation [60], porogen leaching [61], freeze-drying [62] and textile structures [63] are methods that have been used to prepare scaffolds from biodegradable blends or composites, for biomedical applications.

The post-treatment (stabilization) of chitosan polymer/fibers using an ethanol–water mixture is recommended for several reasons. First, an ethanol–water mixture works as a non-solvent for charged polysaccharides [64]. For example, chitosan dissolved in 2% acetic acid is insoluble in a 40% ethanol–water mixture [65]. Second, using ethanol–water (70–30 wt %) produces a perfectly preserved morphology of pure chitosan [64]. Also, it is necessary to prevent the solubilization of chitosan due to the net charge that chitosan has in its salt form: this is particularly important in biomedical applications [65]. To prevent solubilization, the use of an acid with a low boiling temperature (such as acetic acid, 118.1 °C; or formic acid, 100.8 °C) is recommended to allow the extraction of the excess acid and water constituting the solvent that is left after the casting and drying processes. On the other hand, it also necessary to consider the effect that the choice of solvent used in chitosan solvation has on the physical characteristics of chitosan or chitosan composites/blends, for example, the pH, ionic strength, ionic charge and so forth [64,65,66,67,68]. This solvent effect (on the physical characteristics of the formed polymer solution) is illustrated in a previous study on the characterization of chitosan solubilized in aqueous formic and acetic acids: the intrinsic viscosity of chitosan solution in aqueous formic acid is around four times less than the intrinsic viscosity of chitosan in aqueous acetic acid (6.72.3 versus 28.21 cm^3^∙g^−1^) [66]. This difference in solution viscosity is reflected in the shape and properties of the formed polymer/films/membranes from both solvents, as illustrated in our study [66].

In this study, chitosan was selected to be the base material due to its known antimicrobial properties. However, to mitigate its drawbacks mentioned earlier, gelatin was selected both as a blending material to decrease the content of chitosan and as a porogen to create pores by being dissolved out in ethanol–water solution (70–30 wt %). To ensure formation of stable films, ferulic acid was selected as a crosslinker. It was also selected for its natural antioxidant properties, which make it very safe for biomedical applications. Moreover, glycerin was selected to be a natural plasticizer for the prepared nature (green) film/membrane. 

The aim of this study is to investigate a novel, simple method for preparing a chitosan–gelatin film/membrane (polymer matrix) suitable for biomedical applications using acetic or formic acid solvents. The effect of the solvent (acetic or formic acid) used in polymer solution and its effect on the shape and properties of the produced films/membranes will be highlighted. Ferulic acid was used as a crosslinker in the chitosan–gelatin blend. Thermal, spectroscopic and mechanical properties of the chitosan–gelatin–ferulic acid plasticized and non-plasticized film/membrane (polymer matrix) were evaluated to determine if they are suitable for future testing for biomedical applications.

## 2. Experimental

### 2.1. Chemicals

**Figure 1:**
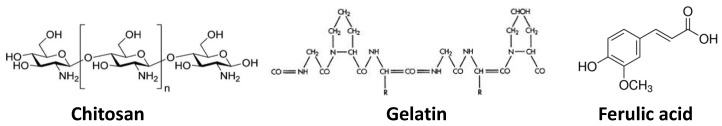
Chemical structure of chitosan, gelatin and ferulic acid.

Chemicals were obtained from the following sources: Chitosan (*M*_w_ 100,000–300,000), ACROS (Geel, Belgium); Gelatin from bovine skin, type B, Sigma Aldrich (Munich, Germany); Ferulic acid (≥99.5%), Fluka (Schwerte, Germany); Glycerin (>98%), Oxford Laboratory Reagent (Mumbai, India); acetic acid (99.9%), formic acid (85%) and ethyl alcohol (95%), Laboratory Chemicals (Cairo, Egypt). Chemicals were used as received. Distilled water was used in all the experiments. The chemical structure of chitosan, gelatin and ferulic acid is shown in Figure 1.

### 2.2. Film Preparation

The chitosan films were prepared using the solvent evaporation technique. Chitosan and gelatin solutions were prepared separately by dissolving 2% (*w/w*) polymer (chitosan or gelatin) in 1 wt % acetic acid or 1 wt % formic acid solution at 50 °C for 1 h. Of each solution, 50 mL were mixed at 50 °C for 1 h. Ferulic acid was added as a crosslinker in specific amounts (1.5–6 mM) and mixed at 50 °C for 1 h. The film-forming solutions were left standing for 2 h at 40 °C, then were poured on to preheated glass petri-dishes and left to dry for 24 h at 40 °C. To plasticize the film, glycerin (0.15 mL, 0.02 wt %) was added to the film-forming solution and mixed at 40 °C for 1 h, before pouring as previously described. The dried films/membranes were peeled off the plate by soaking in ethanol (80%) for 30 min, then the films were left to dry overnight in the atmosphere for characterization.

### 2.3. Film Characterization

#### 2.3.1. Film Thickness

The thicknesses of the chitosan films were determined using a Mitutoyo micrometer (PB-1 JIS.B.7502, Ann Arbor, MI, USA), the average value was calculated from 10 measurements of two different films/membranes (5 measurements per film) that were prepared from two independent polymer solutions for each condition.

#### 2.3.2. Water Uptake

The water uptake (wt %) of the prepared films (scaffolds) was determined as follows: the weights of the samples were determined after drying for 24 h in an oven at 50 °C. The samples were then completely soaked in a specific amount of deionized water for the absorbing process for 3 h. The swollen films were taken out of the water and the excess water on the surface was wiped off with tissue paper until a constant weight was obtained. The water uptake, wt %, of films was calculated as the difference between the wet and dry weights relative to the dry weight (see Equation (1)). Four samples from two films/membranes, which were prepared from two independent polymer solutions, for each condition were used.

(1) $$\text{Water Uptake}\;\left(\%\right)=\frac{\text{Wet weight}-\text{Dry weight}}{\text{Dry weight}}\times 100$$

#### 2.3.3. Static Water Contact Angle 

The static angle of water molecules on each prepared film was determined using a Krüss DSA 100 apparatus (Hamburg, Germany). Drops of demineralized water (7 µL) were deposited on different spots on each film. Four different spots on each of the two different independently prepared films/membranes were measured and results were averaged. All the tested membranes were kept in desiccators for 48 h before measuring.

#### 2.3.4. Fourier Transform Infrared Spectroscopy (FT-IR) Analysis

A Fourier transform infrared spectrometer (Shimadzu FTIR-8400 S, Kyoto, Japan) was used to characterize the prepared pure 2 wt % chitosan and chitosan–gelatin (1:1 g; *r* = 0.5) with and without the ferulic acid crosslinker. The FTIR spectra of the prepared films/membranes were recorded with an attenuated total reflectance (ATR) accessory. The IR spectra were recorded in the wavenumber range of 4000–400 cm^−1^.

#### 2.3.5. X-ray Diffraction (XRD) Analysis

X-ray diffraction patterns of the polymeric films/membranes made with 1 wt % formic acid (pure 2 wt % chitosan and chitosan–gelatin (1:1 g; *r* = 0.5), with and without 3 mM ferulic acid crosslinker and also with added glycerin plasticizer) were obtained on a Shimadzu XRD-7000 X-ray (Kyoto, Japan) diffractometer using a CuKα radiation source operating at 40 kV and 30 mA.

#### 2.3.6. Thermal Gravimetric Analysis (TGA)

TGA analysis of the pure 2 wt % chitosan and the chitosan–gelatin films (1:1 g, *r* = 0.5) with and without 3 mM ferulic acid crosslinker using either 1 wt % acetic acid or 1 wt % formic acid was carried out using a Thermal Gravimetric Analyzer (Shimadzu TGA-50, Kyoto, Japan). The samples were scanned over a temperature range from 20 to 650 °C at a temperature gradient of 10 °C·min^−1^ under nitrogen flow.

#### 2.3.7. Differential Scanning Calorimetry (DSC) Analysis

The pure 2 wt % chitosan and chitosan–gelatin (1:1 g, *r* = 0.5) with 3 mM ferulic acid crosslinker films/membranes were analyzed with differential scanning calorimetry (DSC, NETZSCH, DSC-200PC, Tamil Nadu, India) at a heating rate of 10 °C·min^−1^. The samples were kept in a desiccator before analysis.

#### 2.3.8. Tensile Strength Determination

Samples were cut into a dumbbell shape. The total length and the gauge length of each tested sample were 37 and 18 mm, respectively. The width at the top and the width at the middle of the sample were 13 and 7.2 mm, respectively. Tensile testing of the films was performed with a Texture Analyzer T2 (Stable Micro Systems, Ltd., Surrey, UK), at a constant crosshead speed of 0.1 mm·s^−1^ until breaking. The tensile strength was calculated from the stress–strain curves that were calculated from the load–elongation curves measured in three samples from two films/membranes prepared from two different dopes for each studied condition.

#### 2.3.9. Scanning Electron Microscope (SEM) Imaging

The pure 2 wt % chitosan and chitosan–gelatin films/membranes (1:1 g, *r* = 0.5) with 3 mM ferulic acid crosslinker using 1 wt % acetic acid and the pure 2 wt % chitosan and the chitosan–gelatin films/membranes (1:1 g, *r* = 0.5) with 3 mM and 6 mM ferulic acid crosslinker using 1 wt % formic acid solvent, were imaged using a Scanning Electron Microscope (JeolJsm 6360LA, Kyoto, Japan). The film/membrane (polymer matrix) samples were coated with Au and were imaged at a voltage of 15 KV; both 1000 and 2000 times magnification were used. The pore size of the different tested films/membranes was determined.

## 3. Results and Discussion

### 3.1. Acetic Acid Solvent

The pure chitosan (1 wt % and 2 wt %), chitosan–gelatin (1:1 g; *r* = 0.5) and chitosan–gelatin (*r* = 0.5) with different concentrations of ferulic acid crosslinker (1.5, 3, 4.5 and 6 mM) were prepared using acetic acid solvent (1 wt %) and were used for characterization. As a primary observation, the prepared chitosan–gelatin films/membranes (1:1 g; *r* = 0.5) were homogenous, pale yellow, transparent and slightly brittle in the dry state; whereas, the pure 2 wt % chitosan was rigid and less transparent. The thickness of the prepared pure chitosan increased with increasing chitosan content (Table 1). Replacing half the chitosan content with gelatin did not affect the thickness of the formed film/membrane (which was around 80 µm). Adding the crosslinker ferulic acid resulted in a fluctuation of the film/membrane thickness of 10 µm. Using a glycerin plasticizer did not affect the film/membrane thickness.

**Figure 2:**
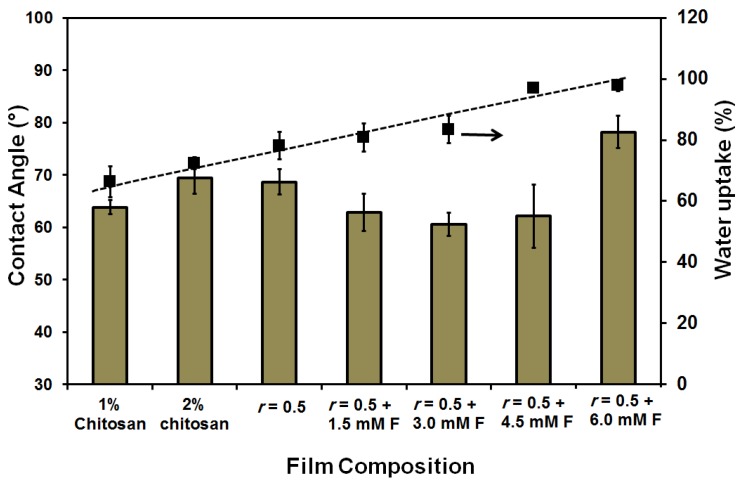
Contact angle and water uptake (%) of the prepared pure chitosan (1 and 2 wt %) and chitosan–gelatin (1:1 g; *r* = 0.5) blended films/membranes using 1 wt % acetic acid solvent with different concentrations of ferulic acid crosslinker [1.5–6 mM].

**Figure 3:**
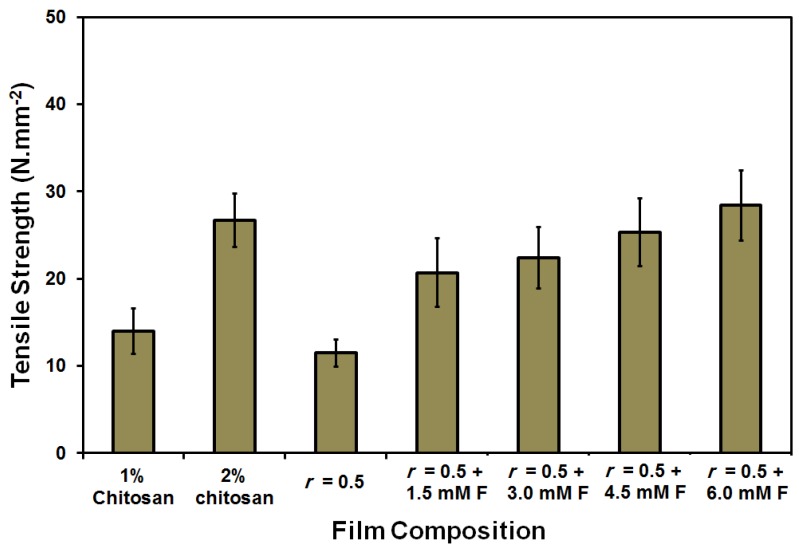
Tensile strength of the prepared pure chitosan (1 and 2 wt %) and chitosan–gelatin (1:1 g, *r* = 0.5) blended films/membranes using 1 wt % acetic acid solvent with different concentrations of ferulic acid [1.5–6 mM].

The water uptake (%) was small (66%) and was gradually increased up to 100% as a result of both increasing the chitosan (2 wt %) content and the addition of ferulic acid crosslinker (see Figure 2). On the other hand, the contact angle slightly fluctuated in the range of 60°–70°. Only in case of using 6 mM ferulic acid, was a higher contact angle (78°) determined. Normally, increasing the crosslinker content resulted in an increase in the contact angle due to both the reduction of the free (polar) groups as the crosslinking process propagates and the formation of a relatively strong network [69], as shown in Figure 3. The replacement of the chitosan with gelatin caused a reduction in film strength to half its original value (from 26.7 ± 3 to 11.5 ± 1.5 N∙mm^−2^). The addition of ferulic acid crosslinker caused a recovery in the strength of chitosan–gelatin (1:1 g; *r* = 0.5) film/membrane to a level that is comparable to the strength of the 2 wt % chitosan film (28 ± 4 N∙mm^−2^). Although the addition of glycerin improves the elasticity of the prepared films (result not shown here), the prepared films/membranes were still relatively rigid and are, therefore, not suitable for biomedical applications such as scaffolding. Films/membranes that are appropriate for medical purposes should have a softness comparable or similar to soft biological tissues [3]. The films/membranes formed using acetic acid can be described as rigid even after adding the plasticizer [3,4].

**Figure 4:**
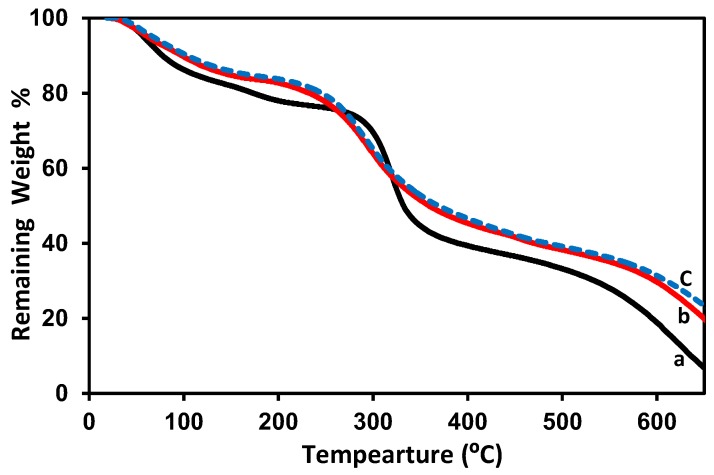
TGA diagram of 2 wt % chitosan (a), chitosan–gelatin (1:1 g; *r* = 0.5) without (b) and with 3 mM ferulic acid (c) blended films/membranes using 1 wt % acetic acid solvent.

Thermogravimetric analysis (TGA) on pure gelatin film/membrane was previously published [60]; the weight loss was determined for the following three ranges: 18–100 °C, 100–420 °C, 420–500 °C. In the first range, of up to 100 °C, a 20% weight loss was measured in the sample, which corresponds to water evaporation. Increasing to 420 °C, resulted in a sample weight loss of approximately 70%, which corresponds to degradation by hydrolysis and oxidation endothermic reactions. Up to 500 °C, 89% weight loss was observed which can be attributed to the pyrolysis of derived collagen. The remaining weight of around 11% consists of ash formed by carbon residues at a temperature higher than 500 °C. TGA of 2 wt % chitosan film/membrane and chitosan–gelatin (1:1 g; *r* = 0.5) film/membrane with and without 3 mM ferulic acid are shown in Figure 4. The initial weight loss at temperatures up to 100–120° C is due to water content removal. The second weight loss occurs in the range of 100–300 °C and may be attributed to the decomposition of the amine groups. The third weight loss occurs in between 300 and 400 °C and may be related to the decomposition of the –CH_2_OH groups [63,70,71]. The decomposition of the chitosan rings continued up to 650 °C until a nearly complete decomposition. The chitosan–gelation (1:1 g; *r* = 0.5) blend showed similar decomposition ranges but with higher remaining weights at each temperature. About 22% remaining weight at 650 °C may be considered as an indication of the formation of a stronger network due to the reaction between the chitosan and gelatin [59,71]. The addition of ferulic acid did not show a change in the performance of chitosan–gelatin (1:1 g; *r* = 0.5).

**Figure 5:**
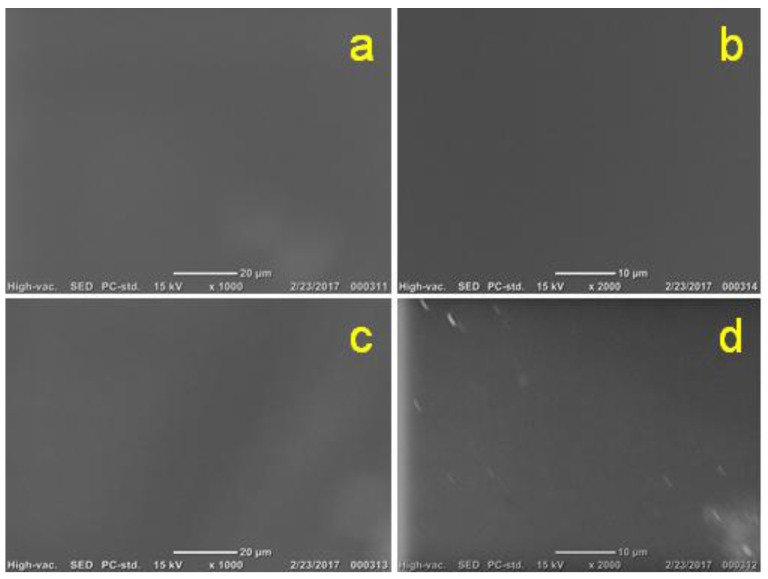
SEM of the pure 2 wt % chitosan at 1000× (**a**) and 2000× magnifications (**b**) and the chitosan–gelatin–ferulic acid (1:1 g; *r* = 0.5, 3 mM ferulic acid) blended films/membranes using 1 wt % acetic acid solvent at 1000× (**c**) and 2000× magnifications (**d**).

SEM of both pure 2 wt % chitosan and chitosan–gelatin (1:1 g; *r* = 0.5), with 3 mM ferulic acid, films/membranes has illustrated the formation of typically extended nonporous (solid) surfaces without any observed difference (Figure 5). These solid surfaces are not promising for use in biomedical applications because they will not help the growth of cells and are expected to undergo degradation difficulties after a short period [69,70,72,73].

### 3.2. Formic Acid Solvent

Using formic acid solvent resulted in films/membranes with much less transparency than the films/membranes prepared using acetic acid solvent. The chitosan–gelatin–ferulic acid films/membranes prepared using formic acid solvent seem to form a polymer coacervation system [74,75]. This may be related to the ability of the ferulic acid to be a crosslinker between the chitosan and the gelatin, forming a very tiny polymer coacervation system (i.e., chitosan–gelatin coacervation occurs through electrostatic interaction) [74,75]. This system can be imagined as having chitosan as the outside frame with the gelatin as the contained component, which may lead to the formation of the open structure seen in SEM images (see next sections) after leaching the gelatin out of the system. In other words, there is a component (i.e., the gelatin-rich phase in which it is possible for chitosan to bind to gelatin) that was leached out by the ethanol solution (80%) that was used to peel the film/membrane off from the petri-dish (i.e., the ethanol–water solution has three roles: solidification of the chitosan-rich phase, leaching out of the gelatin-rich phase, pulling off the film from the glass petri dish). This ethanol solution may leave behind the open-pore network that appears in the SEM images. The high content chitosan (2 wt %) film/membrane that was produced using formic acid solvent was thicker than the film/membrane which was formed using acetic acid solvent. The use of gelatin instead of the half chitosan content resulted in a reduction in the film/membrane thickness to a value comparable to that of the 1 wt % chitosan (Table 2).

The effect of ferulic acid crosslinker was pronounced in the film/membrane thickness in this case (formic acid solvent) relative to the case of using the acetic acid solvent. Most probably, the crosslinking occurs as a result of the ionic interaction between the carboxylic groups of the ferulic acid and the amino groups of chitosan [76]. Also, some of the gelatin may be bonded with the chitosan [52]. The crosslinker reduced the film/membrane thickness from around 58 µm to around 52 µm, whereas using the plasticizer did not affect the measured thicknesses.

**Figure 6:**
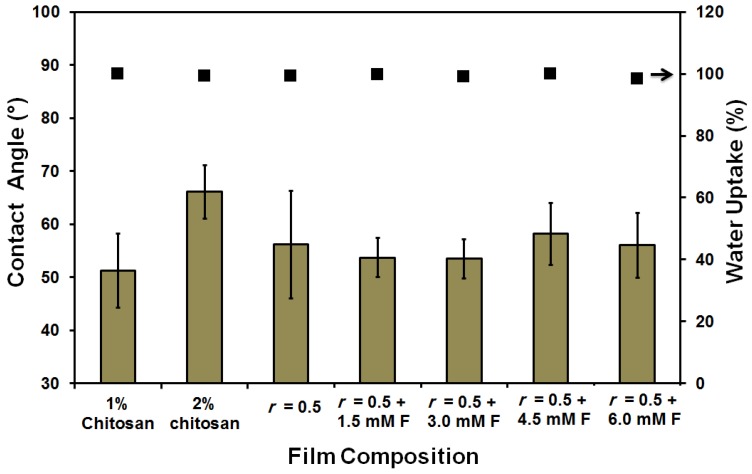
Contact angle and water uptake (%) of the prepared pure chitosan (1 and 2 wt %) and blended films/membranes (1:1 g; *r* = 0.5) using 1 wt % formic acid solvent with different concentrations of ferulic acid [1.5–6 mM].

The water uptake (%) was 100% for all the prepared films/membranes as shown in Figure 6. This is a logical result of the porosity of the film. Also, there was no specific trend in the measured static water contact angles. Again, all the prepared films/membranes showed a fluctuation in the contact angle of around 56° compared to 66° in the case of films/membranes made with acetic acid solvent, whereas the 2 wt % chitosan film/membrane showed 66.1° compared to 69.4° in the case of using the acetic acid solvent. The obtained data can be related to the formation of films/membranes with higher hydrophilicity than those formed using acetic acid solvent. Also, the effect of the porosity of the films cannot be neglected. This hydrophilicity may be attributed to the formed homogenous blend in acetic acid solvent and the indication of bonded networks forming with the possibility of a decrease in the (free) polar groups on the surface [69,70]. The formed blends, in the case of the formic acid solvent, may have produced less bonded networks while keeping free polar groups (as proved by the different analysis techniques) on the formed films/membranes. Also, acetic acid is a weaker acid than formic acid because of both the effects of the methyl group on the stabilization of the dipolar resonance form of the acid and the partial positive charge on the carbonyl carbon that resulted in the destabilization of the resultant carboxylate anion [70]. In previous work [6], it was shown that the equivalent conductivity of chitosan solvated in 0.02 M acetic acid solution was lower than its equivalent conductivity in formic acid solution [69]. This can be attributed to the presence of freer polar groups on the chitosan when formic acid solvent was used. In the present study, the determined pH of the polymer blend solution was 2.57 ± 0.3 when the formic acid solvent was used but when acetic acid solvent was used the pH was 3.95 ± 0.3. The change in the pH of the polymer solution could affect the charging of the components and result in differing characteristics of the formed films/membranes [69,70,71].

**Figure 7:**
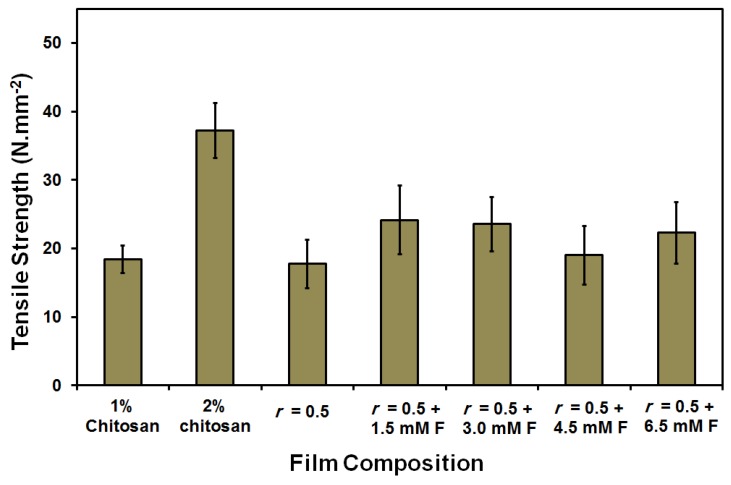
Tensile strength of the prepared pure chitosan (1 and 2 wt %) and blended films/membranes (1:1 g; *r* = 0.5) using 1 wt % formic acid solvent with different concentrations of ferulic acid [1.5–6 mM].

On the other hand, the addition of ferulic acid crosslinker resulted in an increase in the strength of the chitosan–gelatin film/membrane (1:1 g, *r* = 0.5) from 17.7 N∙mm^−2^ to around 24 N∙mm^−2^. However, increasing the used amount of ferulic acid did not cause a significant change in the measured strength; the lowest used concentration in this study had a strength of 1.5 mM F (Figure 7).

Both the chitosan and gelatin were dissolved in formic acid solvent, thus causing the two components to have similar isoelectric points—being net positively charged at a pH of 6.5. An interaction has occurred between the anionic gelatin biopolymer and the cationic chitosan: in particular, the –COOH, –NH and –OH groups on the amino acids in the gelatin and the –OH and –NH_2_ groups of the chitosan. Electrostatic interactions have also occurred. The effect of these reactions was pronounced in the obtained results of the following characterization techniques.

**Figure 8:**
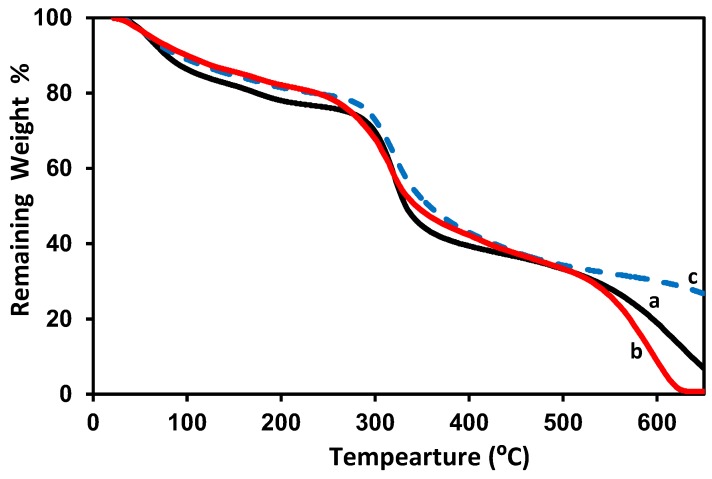
TGA diagram for pure 2 wt % chitosan (a), chitosan–gelatin (1:1 g; *r* = 0.5) film/membrane without (b) and with 3 mM ferulic acid crosslinker (c) using 1 wt % formic acid solvent.

TGA of 2 wt % chitosan, chitosan–gelatin (1:1 g; *r* = 0.5) blend films/membrane without and with ferulic acid crosslinker using formic acid solvent are shown in Figure 8. The four decomposition ranges 100–120 °C, 100–300 °C, 300–400 °C and 400–650 °C are shown for pure 2 wt % chitosan as previously described in the case of acetic acid solvent in Figure 4. The chitosan–gelatin (1:1 g; *r* = 0.5) blend films/membranes without ferulic acid crosslinker, Figure 8b, demonstrated the same performance as pure chitosan but with a higher remaining weight at the same temperature range, although complete decomposition occurred at lower temperature than the decomposition temperature of the pure chitosan (about 610 °C). The addition of ferulic acid crosslinker is shown in Figure 8c: the crosslinking process supports the film/membrane to resist heating, thus maintaining 27% of its weight at 650 °C. The effect of the crosslinker is more pronounced when formic acid solvent was used, compared to when acetic acid solvent was used. In other words, the adding of ferulic acid crosslinker does not improve the resistance of the blend prepared with acetic acid solvent to heat decomposition (see Figure 4a,c), whereas adding ferulic acid crosslinker did indeed improve the resistance of the blend that was prepared using formic acid solvent (see Figure 4a,c). On the other hand, when acetic acid solvent was used, the chitosan–gelatin (1:1 g; *r* = 1) film/membrane with and without ferulic acid crosslinker is stronger than the pure 2 wt % chitosan film/membrane (Figure 4a), while when formic acid solvent was used, the chitosan–gelatin (1:1 g; *r* = 1) film/membrane without ferulic acid crosslinker is weaker than the pure 2 wt % chitosan film/membrane and becomes stronger than the pure 2 wt % chitosan film/membrane after adding the ferulic acid crosslinker. This weakness may be due to the formation of less connected proposed complex/polymer coacervation systems before the solidification process, relative to the solid extend chitosan or the chitosan–gelatin blend when acetic acid solvent was used [74,75].

**Figure 9:**
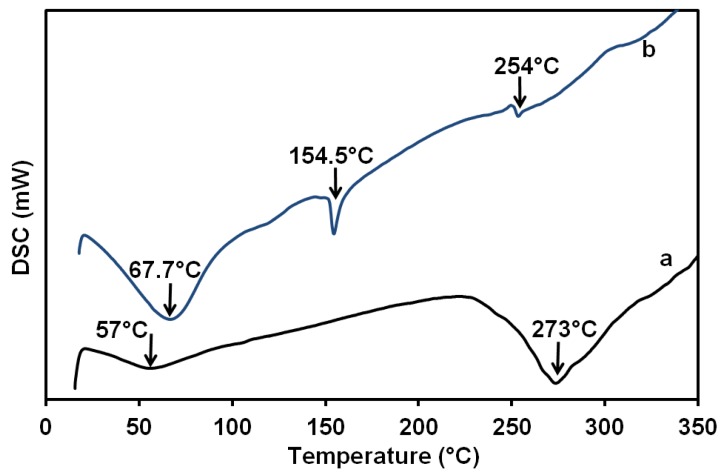
DSC diagram for pure 2 wt % chitosan (a) and chitosan–gelatin–ferulic acid (1:1 g; *r* = 0.5 and 3 mM ferulic acid) film/membrane (b) using 1 wt % formic acid solvent.

The DSC analysis is shown in Figure 9, where the pure 2 wt % chitosan film/membrane (Figure 9a) exhibited an endothermic peak centered at around 57 °C. This peak may be attributed to the loss of water associated with the hydrophilic groups of the chitosan. This may be due to some bound water that was still not removed by drying in the desiccator, which was confirmed by the results obtained from TGA and FTIR analysis. The exothermic peak which appears in the temperature range from 230 to 350 °C and centered at around 273 °C corresponds to the decomposition of the chitosan [77,78]. The chitosan–gelatin–ferulic acid (1:1 g; *r* = 0.5, 3 mM ferulic acid) without plasticizer is shown in Figure 9b. A new decomposition at 154.5 °C is attributed to the melting of ferulic acid crosslinker. The decomposition of non-bound water is overlapped with the decomposition of gelatin to be at a slightly higher temperature range centered at around 67.7 °C. Upon the reaction of the chitosan with lower glass temperature gelatin, its decomposition temperature was reduced to be at 254 °C with much reduction in the peak intensity.

**Figure 10:**
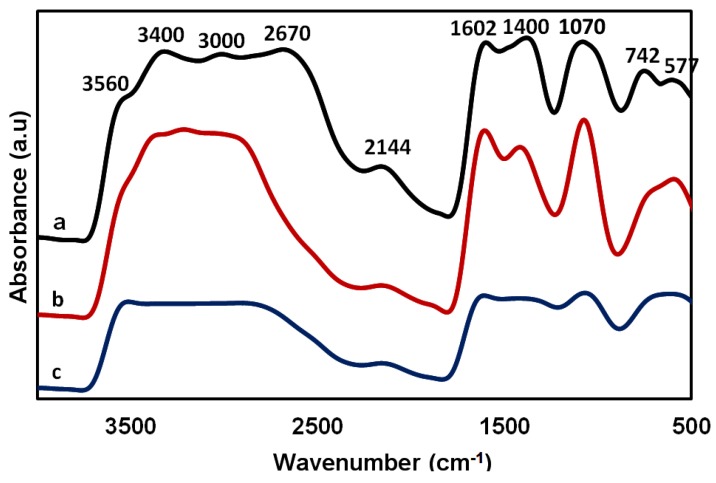
FTIR spectrum of 2 wt % chitosan (a), chitosan–gelatin (1:1 g; *r* = 0.5) film/membrane without (b) and with ferulic acid crosslinker (c) using 1 wt % formic acid solvent.

In Figure 10, the characteristic peaks of chitosan were located between 2670 and 3400 cm^−1^ for the hydroxyl group stretching and 3560 cm^−1^ for the C–N stretching primary amine. The 1602 and 2144 cm^−1^ peak may be related to the amide II and III bands of chitosan, respectively. The spectrum at 1070 cm^−1^ can be attributed to the saccharide band in the chitosan film/membrane (Figure 10a), the chitosan–gelatin film/membrane (Figure 10b) and in the cross-linked case (Figure 10c) where it can be seen to be diminished. This diminishing that occurs with crosslinking may confirm the involvement of the hydroxyl and ether groups of chitosan in the crosslinking of the chitosan–gelatin–ferulic acid film/membrane. Moreover, a band at 1400 cm^−1^ in chitosan and in the spectrum of the chitosan–gelatin film could be attributed to the –COO^−^ group. The peaks at 742 and 2670 cm^−1^ may be related to C–H bending and stretching, respectively [79].

**Figure 11:**
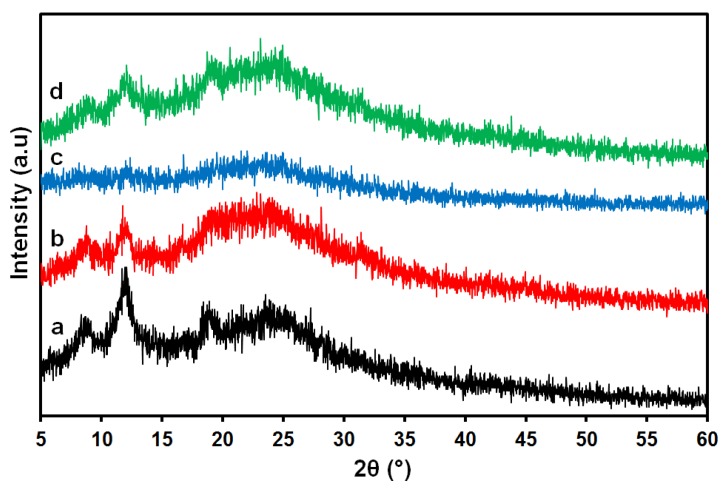
XRD spectrum of 2 wt % chitosan (a), chitosan–gelatin blended film (scaffolds) without (b) and with 3 mM ferulic acid (c) and after adding the glycerin plasticizer (d) using 1% formic acid solvent.

The XRD analysis is shown in Figure 11; the 2 wt % chitosan film/membrane shows a broad peak in the range of 20°–25° as well as two other peaks at 8.8° and 12° [80]. This is in full agreement with the assumption that chitosan is a partially crystalline polysaccharide which contains some crystalline forms embedded in the amorphous region. The chitosan–gelatin film/membrane (without ferulic acid) shows a slight change in the chitosan peak as shown in Figure 11b, which can be an indication of a reaction between the gelatin and chitosan. The addition of ferulic acid crosslinker caused peaks to diminish (as shown in Figure 11c), which may be a sign of the formation of a new network with a change in the internal structure of the formed film/membrane upon crosslinking between the chitosan and the gelatin. The intensity of the peaks was enhanced in the film/membrane after adding the glycerin plasticizer, which may suggest the formation of a new interaction that may lead to a slightly crystalline form as shown in Figure 11d.

**Figure 12:**
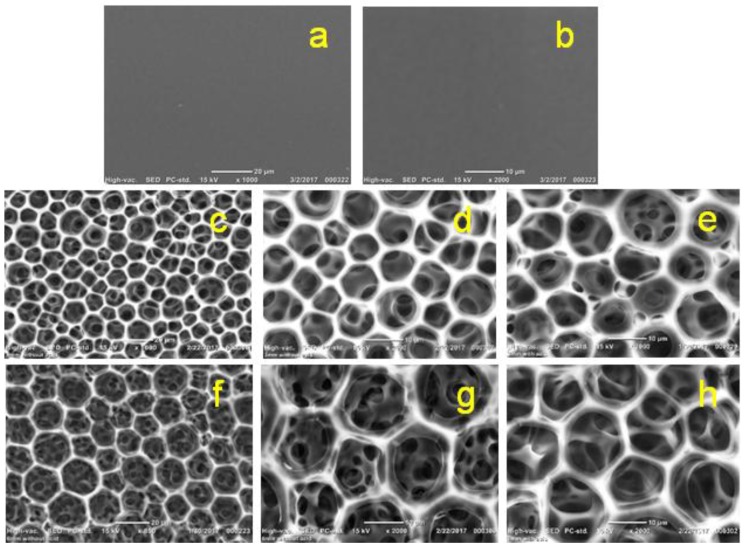
SEM images of 2 wt %. chitosan film/membrane (**a**) 1000× and (**b**) 2000×; chitosan–gelatin blended (1:1 g; *r* = 0.5) films/membranes with 3 mM ferulic acid (**c**) 1000×; (**d**) 2000×; and (**e**) with glycerol plasticizer at 2000×; chitosan–gelatin blended (1:1 g; *r* = 0.5) films/membranes with 6 mM ferulic acid (**f**) 1000×; (**g**) 2000×; and (**h**) with glycerol plasticizer at 2000×.

The SEM imaging of the prepared pure 2 wt % chitosan and chitosan–gelatin films/membranes with two concentrations of the ferulic acid crosslinker are shown in Figure 12. The pure 2 wt % chitosan film/membrane shows a continuous blocked surface (Figure 12a 1000×; and Figure 12b, 2000×). The chitosan–gelatin film/membrane with either 3 mM ferulic acid (Figure 12c, 1000×; Figure 12d, 2000×; and Figure 12e, with glycerol plasticizer at 2000×), or with 6 mM ferulic acid (Figure 12f, 1000×; Figure 12g, 2000×; and Figure 12h, with glycerol plasticizer at 2000×) show hexagonal porous surfaces. Unexpectedly, the size of the formed porous structures seems to be wider when higher concentrations of ferulic acid crosslinker were used. Although the addition of the plasticizer did not affect the performance of the films/membranes as determined by the analysis techniques used in this study, it did make the film/membrane softer and easier to handle in the peeling off process, as well as producing smoother pore edges as observed in the SEM images. This is particularly when a high concentration of the crosslinker was used (3 mM ferulic acid, Figure 12e; and 6 mM ferulic acid, Figure 12h; both at 2000×). A homogenous distribution of the pores with 10–14 µm pore size was observed for almost all the examined films/membranes.

In a previous study [81], freeze-drying solidification of chitosan–gelatin blend was used to prepare a biomedical film/membrane and the network structure was made through the electrostatic interactions between the ammonium ions (–NH^3+^) of the chitosan and the carboxylate ions (–COO^−^) of the gelatin. However, the produced pores were not orientated as is the case in the produced films in this study. In another study [82], chitosan–gelatin hybrid scaffolds with well-organized architectures and highly porous structures were created by combining rapid prototyping, microreplication and freeze-drying techniques. Also, chitosan–gelatin blend fibers have been produced [83] and their advantages over chitosan and gelatin fibers for fabricating engineered scaffolds were illustrated. In that study, trifluoroacetic acid and dichloromethane were used as solvents and the tensile strength of the produced chitosan–gelatin (*r* = 1) was comparable to the strength of the film/membrane produced using acetic acid solvent in our study (around 37.91 ± 4.42 MPa). Also, chitosan–gelatin hybrid polymer network scaffolds were prepared via the freeze-drying technique by using the ice microparticles as a porogen [84]. The bilayer scaffolds were prepared via contact with a −56 °C lyophilizing plate directly, then lyophilized. While there are many other researchers that have investigated the blend of chitosan–gelation for preparing films/membranes that can be used in different biomedical applications, this is the only study that uses a very simple preparation technique and obtains very organized porous films/membranes.

## 4. Conclusions

Using acetic acid solvent for preparing chitosan–gelatin (1:1 g; *r* = 0.5) films/membranes with ferulic acid crosslinker produced solid (nonporous) films/membranes that are not suitable for biomedical applications. However, the prepared films/membranes, in this study, using acetic acid solvent may be recommended for other applications such as packaging purposes.

Formic acid solvent is about 10 times stronger than acetic acid: acidity (pKa) of formic acid is 3.77, whereas for acetic acid it is 4.76. The interactions between gelatin and chitosan are produced by both electrostatic and hydrogen bonding. The gelatin acts as a porogen to create a well-defined size network. The chitosan–gelatin–ferulic acid films/membranes created using formic acid solvent could form a polymer coacervation system. The ferulic acid crosslinks the chitosan (and possibly some of the gelatin bonded with the chitosan: in other words, it forms gelatin-rich and chitosan-rich coacervation systems). Glycerin was used as a plasticizer to improve the softness of the produced films/membranes. The chitosan–gelatin (1:1 g; *r* = 0.5)–ferulic acid films/membranes made using formic acid solvent produced novel hexagonal porous films/membranes with a thickness of around 60 µm, 10–14 µm pore size and a tensile strength of around 20 N∙mm^−2^. The produced films/membranes using formic acid solvent are recommended for biomedical application. Testing the produced films/membranes for cell growth suitability and/or the application of a cytotoxicity assay should be considered in future.

## Figures and Tables

**Table 1:** Thickness of the prepared films/membranes using 1 wt % acetic acid. Chitosan–gelatin films/membranes (1:1 g; *r* = 0.5).

Film/Membrane Composition	Film/Membrane Thickness (mm)
1 wt % Chitosan	0.06 ± 0.002
2 wt % chitosan	0.08 ± 0.001
*r* = 0.5	0.08 ± 0.001
*r* = 0.5 + 1.5 mM F	0.07 ± 0.001
*r* = 0.5 + 3.0 mM F	0.09 ± 0.001
*r* = 0.5 + 4.5 mM F	0.09 ± 0.001
*r* = 0.5 + 6.0 mM F	0.08 ± 0.001
*r* = 0.5 + 3.0 mM F plasticized	0.08 ± 0.001

**Table 2:** Thickness of the prepared films/membranes using 1 wt % formic acid solvent. Chitosan–gelatin films (1:1 g; *r* = 0.5).

Film/Membrane Composition	Film/Membrane Thickness (mm)
1 wt % Chitosan	0.058 ± 0.001
2 wt % chitosan	0.093 ± 0.001
*r* = 0.5	0.058 ± 0.001
*r* = 0.5 + 1.5 mM F	0.051 ± 0.002
*r* = 0.5 + 3.0 mM F	0.051 ± 0.001
*r* = 0.5 + 4.5 mM F	0.052 ± 0.001
*r* = 0.5 + 6.0 mM F	0.052 ± 0.001
*r* = 0.5 + 3.0 mM F plasticized	0.051 ± 0.001
